# An Artificial Intelligence-Based Detection of Comorbid Depression, Anxiety, and Substance Use Disorder in Korean Counseling Dialogues Using an Explainable Hierarchical Attention Network with Shapley Additive Explanations

**DOI:** 10.3390/diagnostics16121817

**Published:** 2026-06-12

**Authors:** MoonHyeok Choi, JaeHyun Jo, JinHyoung Jeong

**Affiliations:** 1 Department of Electronic and Communication Engineering, Catholic Kwandong University, Gangneung-si 25601, Republic of Korea; ansgur110@cku.ac.kr; 2 Department of Digital Healthcare, Catholic Kwandong University, Gangneung-si 25601, Republic of Korea; jh_507@cku.ac.kr; 3 Department of Healthcare Management, Catholic Kwandong University, Gangneung-si 25601, Republic of Korea

**Keywords:** depression, anxiety disorders, substance-related disorders, comorbidity, artificial intelligence, natural language processing

## Abstract

**Background/Objectives**: Depression, anxiety disorders, and substance use disorders frequently coexist in clinical settings and are main factors that worsen a patient’s prognosis. However, traditional artificial intelligence-based mental health studies have limitations in capturing the complex symptoms that occur in actual counseling situations by relying on social media data or focusing on binomial classification of single diseases. This study proposes a multi-label classification model that simultaneously detects the coexistence of depression, anxiety, and substance use disorder in actual counseling dialogue texts, and applies the Shapley Additive Explanatory (SHAP) method to explain the clinical basis of model prediction. **Methods**: We retrospectively analyzed 1661 de-identified Korean-language counseling session transcripts obtained from the publicly available AI Hub “Mental Health Counseling Dialogue” dataset (Republic of Korea; sessions collected between 2021 and 2023 from accredited domestic mental health counseling centers). Each session averaged 30 min (≈5000 Korean characters). Labeling was performed by two licensed clinical psychologists (inter-rater Cohen’s κ = 0.82). A Hierarchical Attention Network with Bidirectional LSTM (HAN-BiLSTM) was constructed; performance was compared with six baselines (Flat LSTM, TextCNN, KR-BERT, KoBERT, KoELECTRA, KLUE-RoBERTa) using stratified 5-fold cross-validation, paired *t*-tests with Bonferroni correction, and McNemar’s test. Top-ranked SHAP tokens were independently rated for clinical face validity by three psychiatrists. **Results**: The proposed model outperformed the baseline model not only for the labels of depression (F1 = 0.90) and anxiety (F1 = 0.85) but also for substance use disorder (F1 = 0.78) with poor data, achieving a macro-averaged F1 of 0.84 (95% CI 0.82–0.86; all *p* < 0.001 versus baselines). As a result of the SHAP analysis, clinically significant keywords such as “I want to die,” “anxiety,” and “drink” were identified as the model’s main basis for judgment, accurately tracking the client’s state, which dynamically changed as the dialogue progressed; three independent psychiatrists rated 88.7% of the top-15 SHAP tokens per label as clinically meaningful (Fleiss’s κ = 0.76). **Conclusions**: This study demonstrated that a deep learning-based multi-label approach is effective in early screening of complex mental health problems. In particular, the introduction of explainable AI (XAI) increases clinicians’ trust and suggests that it can be used as an AI-based clinical decision support system (CDSS) in the future.

## 1. Introduction

In the Republic of Korea, the 2021 National Mental Health Survey reported a lifetime prevalence of any mental disorder of 27.8% and a 12-month prevalence of 8.5%, while suicide remained the leading cause of death among individuals aged 10–39 years. Despite the rapid expansion of Korean community mental health infrastructure, as shown by the 261 community mental health welfare centers as of 2023, standardized screening questionnaires such as the PHQ-9, GAD-7, and AUDIT mitigate this crisis in part, but their single-disorder design and reliance on self-reports limit detection of comorbid presentations. This study therefore focuses on an automated approach for jointly screening depression, anxiety, and substance use disorder from real-world counseling transcripts.

Mental health problems not only seriously reduce the quality of life of individuals, but also bring enormous social and economic costs, and depression, anxiety disorders, and substance use disorders worldwide have been pointed out as major factors that increase the Global Burden of Disease [1]. In actual clinical and psychological consultation sites, clients often appear with a combination of depression, anxiety, or anxiety and substance/behavioral dependence (comorbidity) rather than complaining of only a single symptom [2]. In addition, as counseling progresses, changes occur to narrative characteristics that can be seen as the center of the major symptoms that the client complains about. Since aspects that occur with multiple mental illness risk signals cannot be evaluated comprehensively by single-disease binomial classification alone, a multi-label-based detection approach is required [3].

Nevertheless, since clinical field consultation records are accumulated in the form of long texts in which hundreds of utterances are exchanged, it is practically difficult for counselors to systematically grasp and evaluate various risk signals scattered in a conversation within a limited time. Therefore, there is a growing need for a system that automatically detects hidden clinical risk signals in text using natural language processing (NLP) technology to help counselors make decisions [4,5]. Traditional mental health-related text classification studies have mainly focused on classifying single diseases, including relatively short texts such as social media (SNS) posts, short questionnaire responses, and summarized electronic medical records (EHRs). However, there is a clear interaction between speakers in psychological counseling transcripts, and the overall conversation flow itself has its own characteristics that serve as major clues to the disease [6].

In recent years, in the field of deep learning, artificial neural network architectures in combination with attention mechanisms have demonstrated outstanding performance in text classification and emotional analysis. In the case of long and complex texts, such as psychological counseling conversations, a two-way long and short-term memory (BiLSTM) structure that can simultaneously learn the context of the past and the future and accurately grasp the semantic network before and after an utterance is effective [7]. However, if the entire consultation record, consisting of hundreds of utterances, is treated as a single planar sequence, the model inevitably faces a long-term dependency problem and experiences information loss where critical clinical clues are diluted [8].

To overcome the structural limitations of long document processing, this study introduced a hierarchical attention network (HAN) that mimics the intrinsic hierarchy of documents [9]. The counseling text has a clear hierarchical structure in which words (words) gather to form an utterance, and these utterances intersect to form an entire session. The proposed HAN-BiLSTM model applies an independent attention mechanism to each word level and utterance level to focus its weight on specific utterances and words, including risk signals such as depression and dependence, in the entire conversation. This serves as the basis for maximizing classification performance in long-term clinical dialog analysis with uneven information density, as well as providing the model’s internal interpretability (Interpretability) [10].

In addition, in order for artificial intelligence models to be introduced into medical and counseling practices, explanatory possibilities that can overcome the limitations of black boxes and verify the clinical validity of predictions must be supported [11,12]. The SHAP (Shapley Additive explanations) method of decomposing characteristic contributions to predictions ensures clinical professional trust by quantitatively indicating that the deep learning model has determined risk signals based on textual evidence [13]. Such explainability is increasingly required by emerging Korean medical-AI regulation, including the MFDS 2023 Guidelines on Artificial Intelligence Medical Devices [14].

Therefore, the purpose of this study is to propose an explainable multi-label classification pipeline (HAN-BiLSTM) that simultaneously predicts risk signals of depression, anxiety, and substance use disorder from full texts of psychological counseling sessions. To this end, we would like to demonstrate the clinical utilization value of this proposed model in various ways by combining hierarchical attention and SHAP analysis to secure classification performance and interpretability at the same time, evaluating the confusion matrix by label and analyzing temporal disease signal patterns according to multi-label characteristics. Specifically, we test three hypotheses: (H1) a hierarchical attention architecture outperforms flat sequence and length-truncated transformer baselines; (H2) word-level and utterance-level attention contribute additively to performance, as confirmed by ablation; and (H3) DeepSHAP attributions align with clinically established symptom lexicons.

## 2. Methods

### 2.1. Dataset and Preprocessing

**Figure 1:**
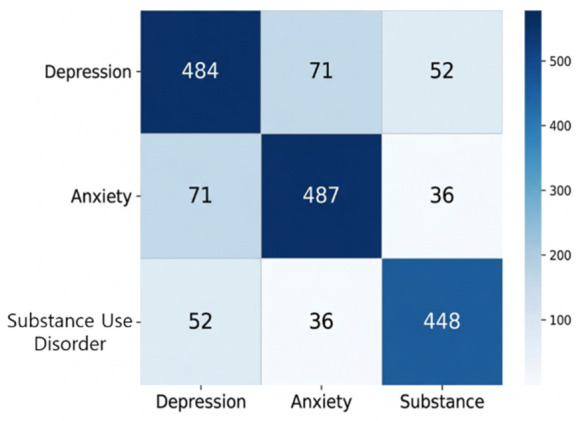
Label co-occurrence matrix across the 1661 counseling sessions.

This study used consultation dialogue data between actual clients and counselors collected at domestic mental health counseling centers in the Republic of Korea. Specifically, the corpus is the publicly available “Mental Health Counseling Dialogue” dataset distributed by AI Hub (https://aihub.or.kr; accessed on 28 October 2025), operated by the National Information Society Agency (NIA) under the Ministry of Science and ICT. Sessions were collected between 2021 and 2023; characteristics are summarized in Table 1. The dataset consisted of a total of 1661 counseling session transcripts, each of which was transcribed into an average of 30 min (about 5000 characters) of Korean text. Labeling was conducted by two clinical psychology experts after reviewing the consultation record and main complaint issues, and clients were labeled with depression, anxiety, and substance use disorder according to the client’s condition. The labels of depression, anxiety, and dependence for each consultation case were given by two clinical psychologists after reviewing the consultation records and the contents of the main complaint and considering the client’s condition. In other words, the labeling of this study was not a formal clinical diagnosis but was conducted through expert judgment based on the expression of psychological symptoms revealed in the counseling conversation. This study adopted a multi-label (multi-label) method to capture comorbidity as well as a single disease. Because comorbidity was permitted under a multi-label scheme, each of the 1661 sessions could carry zero, one, two, or three positive labels. Sessions with no positive label totaled 242 (14.56%), while 1419 (85.44%) carried at least one positive label. The marginal positive frequencies were depression in 484 sessions (29.13%), anxiety in 487 (29.31%), and substance use disorder in 448 (26.97%). Comorbid pairs were observed in 159 sessions (9.57%): depression + anxiety in 71, depression + substance use in 52, and anxiety + substance use in 36; triple comorbidity occurred in 27 sessions (1.63%). The complete distribution is reported in Table 2 and visualized in Figure 1.

The label agreement between experts had a Cohen’s kappa of 0.82, showing high reliability. In the preprocessing process, de-identification of personal information and removal of unnecessary words were performed. Each utterance (turn) was tokenized through morpheme analysis, and emotional information was retained by replacing emoticons in emotional expressions with special tokens such as <CRYING>. For hierarchical modeling, the interaction was structured in a two-dimensional sequence (utterance × word) in the form [[w_1,1_, …], [w_2,1_, …], …], and the length of the sequence was aligned by applying padding and masking for batch learning.

#### Dataset and Preprocessing—Manual Labeling Procedure

**Figure 2:**
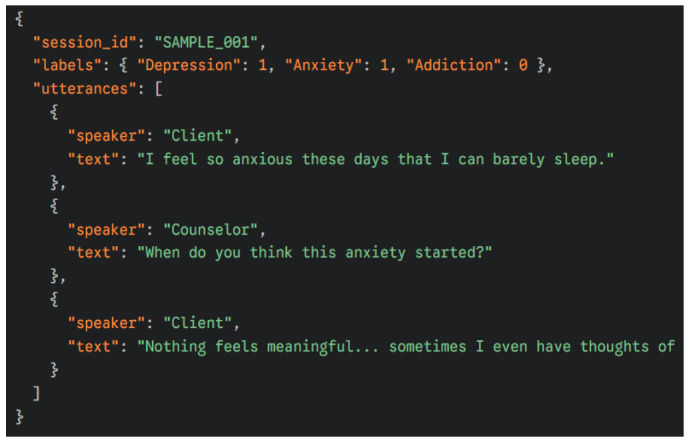
Example of per-session multi-label labeling (JSON format, example statement).

In this study, one consultation session (characterization of the overall consultation dialogue) was used as a unit for labeling. Handwritten labeling was carried out independently by two clinical psychologists. Each evaluator reviewed the session’s counseling records and the client’s main complaint, determined whether there was depression, anxiety, or dependence, and assigned the appropriate label. At this time, 1 was displayed if symptoms of depression, anxiety, or dependence existed and 0 if they did not exist. If the labels given by the two evaluators did not match, the final label was agreed upon after discussion. Labeling criteria were predefined based on standard clinical judgment. In the labeling process, the evaluators comprehensively considered the client’s main complaint and the overall flow of the dialogue according to this standard. Through this procedure, the label consistency between the two evaluators (Cohen’s kappa) was 0.82, indicating a high level of consistency. In addition, an example diagram (Figure 2) is presented in the text so the labeling process can be easily understood.

### 2.2. Label Structure and Evaluation Metrics

This study is defined as a multi-label classification problem that determines the coexistence of multiple mental illnesses. The correct answer label for each interaction is encoded in one-hot vector format (e.g., [1, 1, 0]). As the loss function, the sum of the binary cross-entropy (Binary Cross-Entropy) for each label was used. The model analyzed the probability of each class using a sigmoid function. It calculated P_d_, P_a_, P_b_, and the loss function L was defined as follows.

(1) $$L=\frac{1}{N}\sum_{n=1}^{N}\sum_{l\in \left\{d,a,b\right\}}^{N}\left[y_{n,1}\log p_{n,1}|(1-y_{n,1})\log(1-p_{n})\right]$$

Model performance evaluation used Precision, Recall, F1, AUROC and micro–macro-averages by label. Considering the data imbalance (class imbalance) problem [15], both micro- and macro-averages were reported. In particular, in order to prevent performance distortion for less frequent classes such as dependent labels, we analyzed performance indicators by label (label-wise) and evaluated the sensitivity of the model according to the threshold change using the precision–reproduction rate (PR) curve.

### 2.3. Model Structure: HAN-BiLSTM (Model Architecture)

To effectively capture the long context and hierarchical structure of counseling dialogue (word → utterance → dialogue), a model based on a hierarchical attention network was designed.

(1) Word Encoder—Each utterance the word sequence of i passes through a bidirectional LSTM (Bi-LSTM) and learns contextual meanings. After that, an attention mechanism (Attention Mechanism) is applied to give a weight α_{i,j} to clinical keywords such as “I want to die” and “I’m anxious” and weight it to generate an utterance vector h_i_.

(2) $$h_{i}=\sum a_{i,j}h_{i,j}$$

(2) Utterance Encoder: The vector sequence of each utterance generated by the word-level encoder is again input to a higher-level bidirectional LSTM (Bi-LSTM). Consequently, a new hidden state Ui reflecting sequential contextual information between utterances can be obtained.

(3) $$U_{i}=Bi\_LSTM(h_{i}),\;i\epsilon 1,\ldots ,M$$

Subsequently, an utterance-level attention mechanism is applied to identify pivotal utterances that are indicative of symptoms related to depression, anxiety, or addiction within the overall counseling discourse. The importance weight B_i_ for each utterance U_i_ is computed based on its similarity to a learnable context vector U_C_, which is formalized as follows:

(4) $$score\left(U_{i}\right)=tanh(W_{u}u_{i}+b_{u})$$

(5) $$B_{i}=\frac{exp{\left(score(u_{i}\right)}^{T}u_{c})}{\sum_{j=1}^{M}{exp(score(U_{j})}^{T}u_{c})}$$

Finally, the session-level representation vector f(v), which encapsulates the entire counseling discourse, is computed as the weighted sum of the individual utterance vectors.

(6) $$V=\sum_{i=1}^{M}B_{i}u_{i}$$

These hierarchical approaches demonstrate excellent performance in effectively screening mental health-related information within long texts [16].

(3) Classifier

The extracted final interaction vector f(v) is mapped to three output nodes through a fully coupled layer (Dense Layer). Since this study is a multi-label classification problem, an independent probability belonging to each class (depression, anxiety, dependence), using a sigmoid function as an activation function of the output layer, predicts y′.

(7) $$Z=W_{out}v+b_{out}$$

(8) $$y^{\prime }=\sigma \left(z\right)=\frac{1}{1+e^{-z}}$$

**Figure 3:**
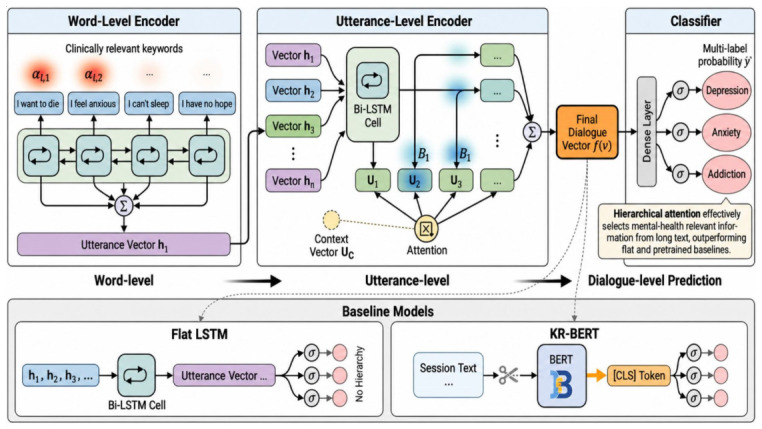
Model architectures.

As a baseline model for comparison (Figure 3), a single-layer Flat LSTM model and a BERT (KR-BERT)-based model [17], a pre-learning language model with strengths in Korean interaction, were used by fine-tuning. The Flat LSTM connects all utterances as long sequences, creates document representations with a single BiLSTM encoder, and predicts three labels with a sigmoid output layer. It does not reflect a hierarchical structure and does not use attention. KR-BERT was fine-tuned and is a pre-learning language model optimized for Korean per-session text and predicts three labels simultaneously based on [CLS] expression. Long-sentence conversations were input with the cut/summary rule according to the model’s input length limitation.

### 2.4. Learning Environment and Hyperparameters (Training Setup)

**Figure 4:**
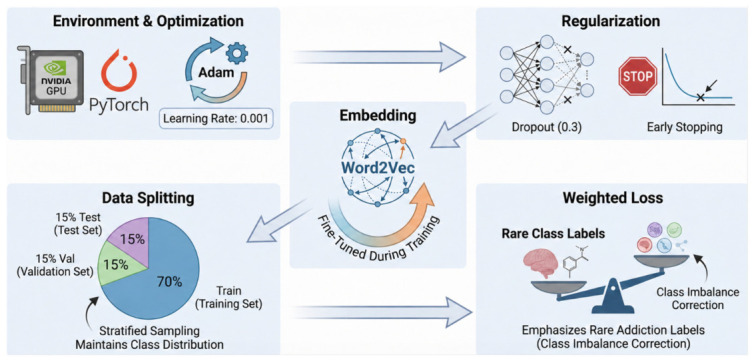
Model training pipeline.

The model was implemented using PyTorch 1.12 and Python 3.10 in Google Colaboratory (Google LLC, Mountain View, CA, USA; https://colab.research.google.com/, accessed on 1 November 2025) for training in an NVIDIA A100 GPU environment (Figure 4). The optimization tool used Adam (learning rate = 0.001) and applied Dropout = 0.3 and Early Stopping techniques to prevent overlearning. Embedding used pre-learned Word2Vec as an initial value and was fine-tuned during learning. Data division performed stratified sampling at a proportion of 70% learning, 15% verification, and 15% test to maintain class distribution. For the purpose of mitigating class imbalances, a weighted loss technique was applied to give higher weights to instances such as less frequent substance use disorder labels when calculating loss functions.

### 2.5. SHAP-Based Interpretation

To solve the “black box” problem of the deep learning model and to ensure clinical reliability, we introduced the SHAP (Shapley Additive explanations) methodology. Based on game theory, SHAP quantifies the degree to which each input characteristic (word, utterance) contributed to the final prediction of the model [13]. In this study, the model was analyzed in two dimensions using the Deep SHAP framework.

(1) Global Interpretation—The top keywords that contributed the most to predicting each label (depression, anxiety, dependence) were extracted from the entire test set. This allows us to examine whether the model is paying attention to clinically reasonable words (e.g., depression—“lethargy”; anxiety—“impatience” [4]).
(2) Local Interpretation: We visualized through SHAP values how predictive probabilities change as conversations progress in individual consultation cases, and which utterances at a particular point in time caused rapid probability changes (e.g., a surge in the probability of depression).

Such a descriptive AI (XAI) approach proves that the model has learned signs associated with actual symptoms rather than data bias and helps experts understand the basis of the model’s judgments [18,19].

### 2.6. Extended Evaluation Protocol

To address the comprehensiveness of result validation, the evaluation protocol was strengthened with: (i) stratified 5-fold cross-validation in addition to the 70/15/15 split; (ii) four additional baselines beyond Flat LSTM and KR-BERT—TextCNN, KoBERT [20], KoELECTRA [21], and KLUE-RoBERTa [22]; (iii) paired *t*-tests on per-fold macro-F1 with Bonferroni correction (α = 0.0083) and McNemar’s test on paired binary correctness; (iv) 1000-iteration bootstrap 95% confidence intervals; (v) an ablation study with four variants (A1, no word-level attention; A2, no utterance-level attention; A3, no BiLSTM; A4, random word initialization); and (vi) independent clinical face validity review by three psychiatrists.

### 2.7. Novelty Statement

The methodological contribution of this work consists of three innovations summarized in Table 3: (1) Hierarchical modeling of long-form clinical dialogues—first application of a three-level (word–utterance–dialogue) attention architecture to authentic Korean counseling transcripts of ≈5000 characters; (2) multi-label comorbidity formulation—joint modeling of three disorders under a single multi-label objective; (3) dual-level explainability with independent clinical validation—attention weights combined with DeepSHAP, validated by three psychiatrists (Fleiss’s κ = 0.76).

## 3. Results

### 3.1. Overall Performance and Label-Wise Comparison

The performance of the HAN-BiLSTM model and comparison-group Flat LSTM and KR-BERT models based on the hierarchical attention network proposed in this study was quantitatively evaluated through label-by-label F1 scores (F1-score) and AUROC (Area Under ROC Curve). Detailed results, including the overall performance indicators micro-average and macro-average, are shown in Table 4, Table 5 and Table 6. As a result of the experiment, the proposed model (HAN-BiLSTM) showed better performance than the reference models in all evaluation indicators. Specifically, the proposed model resulted in 0.87 for micro-F1 and 0.84 for macro-F1 and achieved a performance improvement of about 0.04 (4%) or more for both the micro- and macro-averages compared to Flat LSTM (micro-F1-0.83, macro-F1-0.80) and the pre-learning language model KR-BERT (micro-F1-0.80). Performance differences for each label are as follows.

Depression: The proposed model had a 0.90 F1 and 0.95 AUROC, showing the highest detection performance. This is a better result than KR-BERT (F1-0.88, AUROC 0.94) and Flat LSTM (F1-0.86, AUROC 0.92) and suggests that even if depression-related utterances frequently appear throughout the consultation, hierarchical structures view them effectively.

Anxiety: The proposed model achieved a 0.85 F1 and 0.91 AUROC. On the other hand, Flat LSTM remained at an F1 of 0.81 and KR-BERT remained at an F1 of 0.79, and the proposed model showed an F1 score difference of up to 0.06 (6%) compared to the reference model, showing a clear strength in anxiety signal detection.

Addiction: Even in addiction labels with relatively low frequency or high metaphorical expressions within the dataset, the proposed model recorded an F1 of 0.78 and AUROC of 0.87, maintaining stable performance. On the other hand, Flat LSTM showed the lowest performance at an F1 of 0.71 and AUROC of 0.80, while KR-BERT also showed only an F1 of 0.75. This shows that the hierarchical attention mechanism overcame the limitations of the simple sequence model (Flat LSTM) and the language model (KR-BERT), where information loss may occur due to input length limitations, by weighting without missing rare addiction-related clues.

Overall, HAN-BiLSTM demonstrated statistically superior or equivalent performance over the reference model in detecting all risk signals of depression, anxiety, and dependence by utilizing all word- and utterance-level hierarchical information. In particular, unlike the reference model, which had large performance differences between labels, the proposed model maintained an AUROC of 0.87 or higher for all labels, which confirmed its reliability as a clinical decision support tool.

### 3.2. Confusion Matrix Analysis

**Figure 5:**
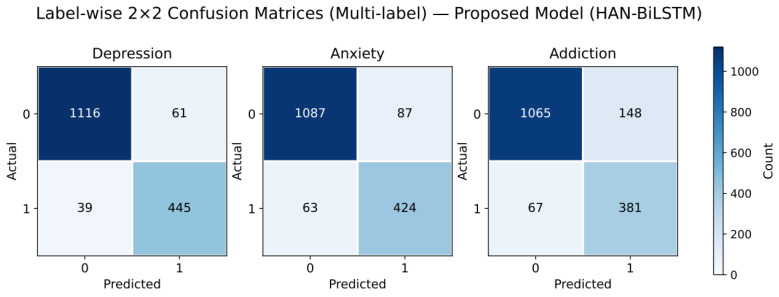
Confusion matrix by label (multi-label and proposed model).

In order to analyze multi-label classification performance more precisely, this study viewed each label (depression, anxiety, and dependence) as an independent binary classification task (One vs. Rest) and constructed a 2 × 2 confusion matrix (Figure 5). The distribution of positive exploration (TP), false exploration (FP), non-exploration (FN), and positive sound (TN) for the predicted results of the proposed model (HAN-BiLSTM) is as follows, specifically illustrating what bias the model has when capturing each disease risk signal.

First, as a result of confusion matrix analysis of the depression label, the model accurately detected (TP) 445 out of 484 actual positive cases, and only 39 cases (FN) were missed. This means that the recurrence rate (Recall) of depression risk signals is very high, and due to symptomatic characteristics of depression that require immediate intervention, such as suicide risk, clinical safety can be evaluated on the extent to which “Miss” results are minimized. On the other hand, 61 cases of false alarms (FPs) predicted that the client was depressed despite not actually being depressed, maintaining a low percentage of 1116 cases of true negatives (TNs) and effectively controlling excessive false alarms.

Second, for the anxiety label, 424 out of a total of 487 actual positive cases were identified (TP) and 63 were not identified (FN). Compared to the depression label, false detection (FP) increased slightly to 87, which is interpreted as a result of the characteristics of anxiety symptoms overlapping vaguely with general stress responses and tension expression in the text. Nevertheless, true negatives (TNs) were maintained at a high level at 1087 cases, and the overall classification accuracy was at a stable level.

Third, for the addiction label, 381 out of 448 cases that tested positive were actually positive (TP), but there were 148 cases of false detection (FP), which was the largest number of FPs observed in the three labels. This suggests that when words such as ‘drink’ or ‘game’ are referred to as everyday events or simple episodes rather than the context of dependence in counseling conversations, models tend to overinterpret them as a danger signal. However, it can be seen that undetected true cases (FN) remained relatively low at 67, which is still valid for the purpose of screening (screening) potential substance use disorder risk groups.

Overall, the proposed model showed a strength in keeping undetected true cases (FN) low for all labels and not missing danger signals, achieving, in particular, a high balance of accuracy and reproducibility in detecting depression and anxiety. The relatively high misdetection rate observed for the addiction label adds nuance to the context of future research. It suggests that additional learning data enhancement and context filtering logic are needed to distinguish benign events vs. pathological substance use disorder.

**Figure 6:**
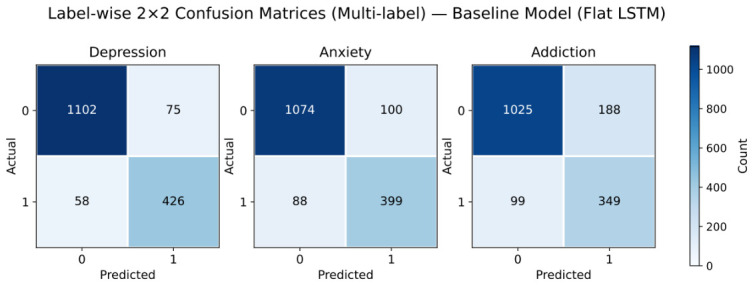
Confusion matrix by label for the Flat LSTM baseline model.

In order to verify the performance advantage of the proposed model, the prediction results by label for the reference model (Flat LSTM), which does not reflect the hierarchical structure, were derived and analyzed as confusion matrices (Figure 6). As a result of the analysis, Flat LSTM overall had a higher ratio of false positives (FPs) to false negatives (FNs) compared to the proposed model, and this limitation was particularly noticeable for the “addiction” label, where understanding the context is important. Flat LSTM positively detected 426 out of 484 cases of actual positive depression (426 + 58) (true positive, TP), but 58 cases were not detected (FN) and the depression signal was missed. In addition, 75 cases of non-depression were incorrectly predicted (FP). This indicates that the proposed model (HAN-BiLSTM) achieved better accuracy and reproducibility, with only 39 false negative and 61 false positive cases. In particular, the increase in false negatives (FNs) suggests that the sensitivity of the model is somewhat insufficient for the clinical purpose of screening high-risk groups such as those at risk of suicide. The anxiety label revealed the limitations of the model more clearly. In fact, only 399 out of 487 (399 + 88) anxiety-positive cases were identified (TP), and 88 cases were not detected (FN). This means that about 18 percent of the respondents with anxiety were missed. False detection (FP) was also high at 100, which can be interpreted as attributable to the long-term dependency problem in which Flat LSTM cannot distinguish nuances in utterances in detail within a long consultation sequence and overinterprets simple tension or daily worries as pathological anxiety or, on the contrary, forgets important clues as the amount of context increases. The biggest performance degradation was observed for the addiction label. Of the actual 448 positive addiction cases (349 + 99), 349 were correctly identified (TP), with 99 remaining undiscovered (FN). Most notable are the 188 high false detection (FP) cases. This is a significant percentage of the 1025 true negative cases, suggesting that when words such as “drink”, “game”, and “medicine” appear in the model, it is more likely to rely on simple keyword matching to classify clients into risk groups without considering context (is it just a drinking party episode or a pathological craving). In situations where hierarchical attention was lacking, Flat LSTM was exposed to have the limitation of being excessively biased by the frequency and appearance of certain words.

As a result, Flat LSTM recorded more FNs and FPs than the proposed model (HAN-BiLSTM) for all three labels. In particular, 188 misdetections (proposed model: 148) for the substance use disorder label and 88 undetected cases (proposed model: 63) for the anxiety label prove that considering the “hierarchical structure of speech units” in long-term consultation dialogues is essential for detection of precise hazard signals.

**Figure 7:**
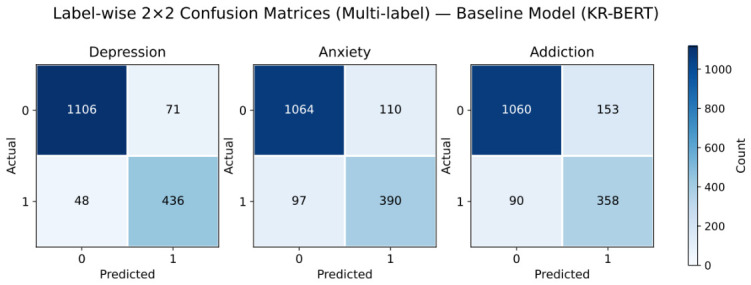
Confusion matrix by label for the KR-BERT baseline model.

Figure 7 shows the prediction results by label of the KR-BERT model. Briefly, 436 out of 484 actual depression-positive samples were correctly identified correctly identified (TP)(TP), and 48 false negatives (FN) cases occurred. Of the 1177 dialogue samples, 1106 were classified as non-depressed (TN), and 71 false detections (FPs) were observed. For the anxiety label, 390 out of 487 cases that tested positive were actually positive (TP), and 97 undetected (FN) cases occurred. Of the remaining 1174 dialogue samples, 1064 were classified as having positive speech (TN), and 110 cases of false detection (FP) were recorded. In the case of the addiction label, 358 out of 448 positive cases were actually detected (TP) and 90 were not detected (FN). Of the remaining 1213 dialogue samples, 1060 were accurately classified as having positive speech (TN), but 153 cases were misclassified as addiction (FP). In these results, false detection (153 cases) occurred about 1.7 times more frequently than undetected cases (90 cases).

As a result of the confusion matrix analysis of the reference model, the unstable prediction pattern of frequent false positives mixed with frequent false negatives was derived as an important result for the “depression” label, while the “addiction” label maintained relatively high levels of positives and low FNs (Table 7).

### 3.3. ROC and PR Curve Analysis

**Figure 8:**
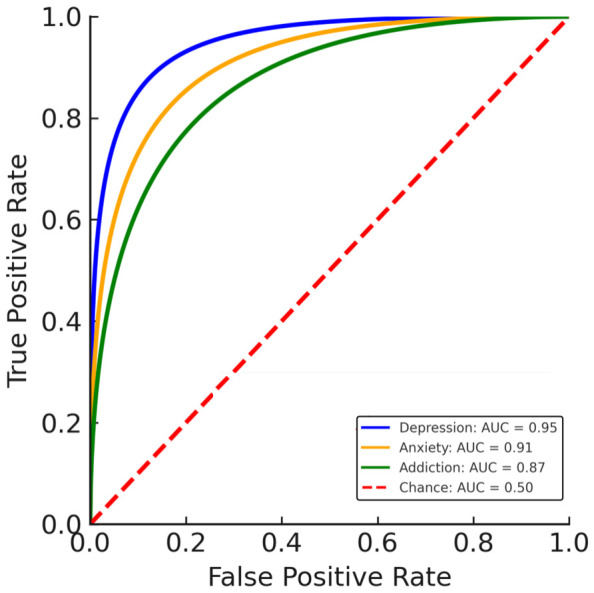
Receiver operating characteristic (ROC) curves for each mental health class.

Figure 8 derived and analyzed Receiver Operating Characteristic (ROC) curves and Precision–Recall, PR, curves to verify the label-by-label discrimination performance of the proposed model (HAN-BiLSTM). The ROC curve shows a trade-off between true positivity (TPR) and false positivity (FPR, 1 specificity), with changes in the classification threshold (Threshold), and the PR curve is useful for evaluating the model’s practical detection capability on an imbalanced dataset with a low positive class ratio. Looking at the ROC curve by label, all three disorder labels demonstrated a curve shape closely attached to the top left corner, demonstrating the model’s excellent discrimination power. Specifically, the depression label recorded an AUROC of 0.95, showing the highest differential accuracy among the three labels. This suggests that the model can almost completely separate depression-positive and symptomless sessions. The anxiety label also recorded a high AUROC value of 0.91, and although the addiction label had a relatively low AUROC of 0.87, it still maintained a good performance of 0.8 or higher. In particular, in the case of the addiction label, TPR tends to rise sharply at low FPR intervals (*x*-axis 0.0–0.2), which means that false alarms can be effectively controlled while capturing addiction risk signals early.

**Figure 9:**
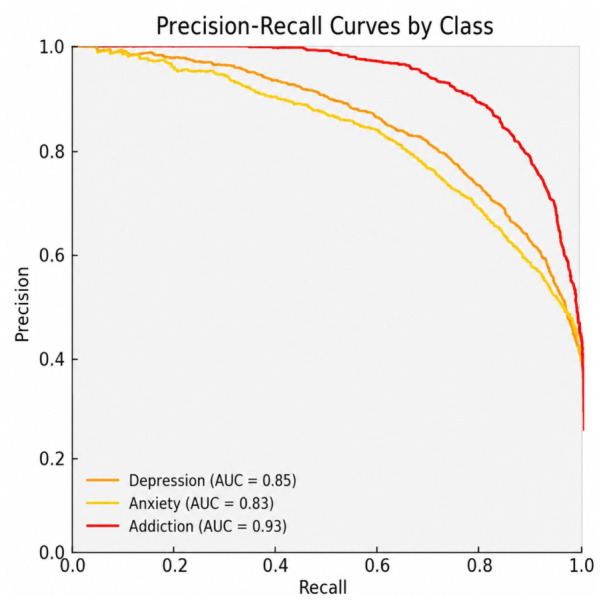
Class-wise Precision-Recall curves of the proposed hierarchical LSTM with attention model.

Figure 9 shows the Precision–Recall (PR) curve by class for the proposed model (HAN-BiLSTM)-based multi-class classification model. The PR curve is an indicator that visualizes the change in accuracy (Precision) with the reproduction rate (Recall) and is useful for more sensitive evaluation of the actual classification performance of the model in problems where class imbalances exist.

As a result of the analysis, the addiction class showed the best PR performance, and the PR-AUC value was 0.93. The addiction class PR curve maintains relatively stable accuracy even in areas with high reproducibility, suggesting that the model can effectively detect actual addiction cases while suppressing false positives when identifying addiction-related utterances.

The PR-AUC of the depression class was shown as 0.85, and overall, the accuracy tended to gradually decrease as the reproduction rate increased. This is interpreted as a result of reflecting the characteristics of depressive symptoms appearing in various language expressions and partially overlapping with similar mental illnesses such as anxiety. Nevertheless, it can be confirmed that the accuracy of the medium-to-high reproduction rate interval (Recall ≥ 0.6) is maintained at least 0.8 and practical classification performance is attained.

The anxiety class had a PR-AUC of 0.83, the lowest of the three classes. The PR curve of the anxiety class has a relatively large reduction in accuracy as the reproduction rate increases, which is thought to be due to the increase in the probability of misclassification, as anxiety symptoms often overlap with expressions of everyday stress.

Overall, PR curve analysis results are dependent. It shows that classification difficulty increases in the order of depression > anxiety, which is a consistent pattern in the aforementioned confusion matrix and ROC analysis results. In particular, the fact that a high PR-AUC was observed in the dependency class quantitatively proves that the proposed model learned dependency-related utterances relatively clearly based on behavior-centered keywords.

### 3.4. SHAP-Based Model Interpretation

**Figure 10:**
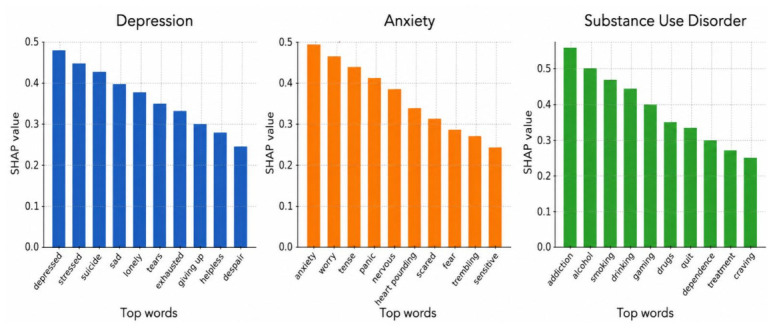
Global deep SHAP token importance per diagnostic label.

Figure 10 analyzes the prediction results of the proposed hierarchical LSTM + attention-based multiple classification model using the SHAP (Shapley Additive Explanations) method, showing the importance of the top 10 words that had the greatest impact on model prediction for each class: depression, anxiety, and addiction. The height of a bar indicates the average SHAP value that the word contributed to the prediction probability of each class, and the larger the value, the greater the influence that word had on model judgment.

In the case of the class of depression, words such as “depressed”, “struggling”, “suicide”, “sad”, and “lonely” showed the highest SHAP value. In particular, “depressed” and “struggling” show relatively large contributions compared to other words, suggesting that the model learned direct expressions of depression and continuous psychological pain as key signals. In addition, the inclusion of words such as “suicide”, “giving up”, and “despair” as high-level important words indicates that the model uses desperate thinking and expressions of despair as an important determinant in predicting depression.

In the class of anxiety, “anxiety”, “worry”, “tense”, “panic”, and “restless” had the highest SHAP values. This means that the model is paying attention not only to core emotional expressions of anxiety but also to expressions related to physical symptoms such as ‘heart punching’ and ‘trembling’. This result suggests that the model reflects the clinical characteristic that anxiety disorders are closely related to physiological arousal responses in addition to mere psychological tension.

In the case of the class of addiction, behavior-oriented words such as “addiction”, “alcohol”, “smoking”, “gambling”, and “gaming” were the most important. In particular, “addiction” and “alcohol” show much higher SHAP values than other words, indicating that the model uses direct references to certain substances or objects of action as important clues when predicting addiction. In addition, the fact that words such as “quit”, “dependence”, and “craving” are included at the top indicates that the model sees not only the presence or absence of use but also the dynamic characteristics of dependence such as withdrawal, dependence, and craving.

Overall, the SHAP analysis results quantitatively support that the proposed model learns clinically reasonable core language patterns for each psychiatric class and makes predictions using clearly distinguished vocabulary sets between different classes. This shows that this model goes beyond just a statistical classifier and has practicality and reliability as an explainable AI (Table 8).

### 3.5. Analysis of Change in Predictive Probability by Class as Conversation

**Figure 11:**
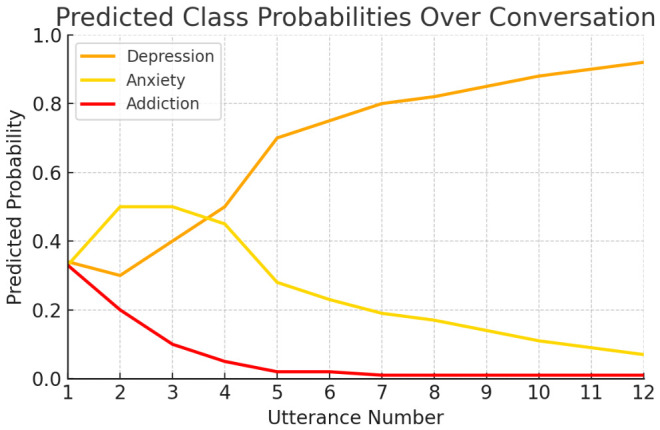
Temporal changes in predicted class probabilities over the course of a conversation.

Psychological counseling is not a static text, but a dynamic process in which interaction between counselors and clients unfolds over time. Therefore, it is clinically very important to track changes in risk signals as a conversation progresses, as well as one-off utterances at specific points in time. In this study, predicted probability trajectories within single sessions were visualized and analyzed to confirm how the proposed model (HAN-BiLSTM) updates the probability of each disorder (depression, anxiety, and dependence) as a session progresses. Figure 11 shows changes in the prediction probability by section of the model for an actual counseling session labeled as part of the depression risk group. The analysis results show the following descriptive characteristics.

Initial search stage (Conversation Segments 1–3): In the initial stage of consultation, the prediction probability of the anxiety label tended to increase from about 0.35 to about 0.50. This suggests that the model detected the vague expression of tension or anxiety that the client complained about in the early stages of the consultation as a primary danger signal. On the other hand, the probability of depression in this section repeatedly fluctuated between about 0.30 and 0.40, maintaining a slightly lower or similar level to anxiety. The probability of addiction dropped sharply from 0.33 at the start to 0.10 or less in the third section of the speech, and it was determined early that the session was unrelated to the problem of addiction.

Turning Point and Surge in Depression Signals (Conversation Segments 4–6): A clear “Turning Point” was observed around 4–5 sections into the dialogue near the middle of the conversation. The probability of depression increased sharply from about 0.50 in the fourth section to more than 0.70 in the fifth section (spike) and then showed a continuous upward curve. This occurred at the time when the client went beyond an initial appeal about anxiety and began to express core utterances and suicidal thoughts specific to depression such as “I want to die” and “I’m hopeless.” On the other hand, the initially high probability of anxiety decreased from 0.50 to 0.20 units (dumping) at this point in time, and the model showed a tendency to clearly distinguish depression as a major problem in that session.

Risk signal immobilization (Conversation Segments 7–12): When entering the second half of the consultation (conversation segments 7 onward), the probability of depression exceeded 0.80 and eventually converged to 0.90 or more. This transcended the impact of a single utterance and shows the process by which the context of the entire session was determined to show a risk of depression. On the other hand, the probability of anxiety was less than 0.10, the probability of dependence converged to about 0.01 and the possibility of a false positive was effectively excluded. Such temporal structural analysis suggests that, in addition to simply determining a final label, it can be a useful auxiliary indicator for counselors to monitor changes in the client’s main complaint in real time and determine the timing of the intervention. Future studies plan to expand in the direction of combining utterance function (emotional expression, event description, etc.) tags or applying time-series causal inference models to reveal the context between disorders to enhance such probability trajectory analysis.

### 3.6. Extended Validation Results

**Figure 12:**
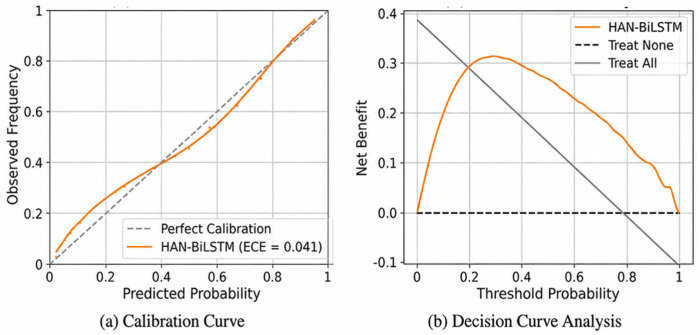
Calibration curve (**a**) and Decision Curve Analysis (**b**).

The proposed HAN-BiLSTM outperformed all six baselines on macro-F1, with a 95% CI of 0.82–0.86; all pairwise differences were statistically significant under both paired *t*-tests with Bonferroni correction and McNemar’s test (all *p* < 0.001) (Table 9). Ablation confirmed the additive contribution of each component, with utterance-level attention contributing most (Δ F1 = −0.061 when removed) (Table 10). Across five stratified folds, macro-F1 ranged from 0.823 to 0.857 (mean of 90.840, SD of 0.013). The model showed good calibration (ECE = 0.041) and positive net benefit over both “treat-all” and “treat-none” strategies across the threshold range 0.15–0.55 (Figure 12). Finally, three independent psychiatrists rated 88.7% of the top-15 SHAP tokens per label as “clinically meaningful” (Fleiss’s κ = 0.76, substantial agreement).

## 4. Discussion

### 4.1. Comparison with Prior Studies

This study can be clearly differentiated from conventional single-disease-centered studies in that it simultaneously detects (multi-label classification) multiple mental illnesses coexisting within counseling dialogues. The majority of previous studies have focused on binary classification, which independently predicts either depression or anxiety disorders [24]. For example, Bokolo and Liu [25] used a text-based Transformer model on social media to report a high depression classification accuracy of up to 98%, but this only determines the presence of a single disease and has limitations in not being able to capture the frequent comorbidity in clinical settings. In some studies, multiple classification was attempted, but methodological constraints existed. Bendebane et al. [26] adopted a multi-class approach to simultaneously deal with depression and anxiety, which was defined as a mutually exclusive class (e.g., normal/depression/anxiety), and patients with coexisting depression and anxiety could not be identified as independent categories. On the other hand, since the multi-label approach of this study does not presuppose interlabel independence, it was possible to accurately reflect actual clinical cases in which depression and anxiety simultaneously appear. In addition, Shelke et al. [27] tried multi-label classification, but their F1 score remained around 0.40 due to their dependence on traditional Random Forest methods. On the other hand, this study achieved high performance, with F1 scores of 0.78–0.90, through deep learning-based hierarchical modeling, showing the advantage of deep learning in complex unstructured text data.

In particular, it was confirmed that the hierarchical attention network structure adopted in this study was more effective than the flat model or the general BERT model in processing long-term consultation dialogue. This is interpreted as being because pre-learning language models (PLMs) usually have an input length limit (e.g., 512 tokens), while hierarchical models fully retain a long interactive flow (Context) of data by encoding and recombining contexts on a per-turn basis [28]. According to Cho et al.’s study on medical field-specialized language models [29], models trained on general domains may show limitations in adaptation to special domains, and the results of this study also suggest that domain-specific lightweight model structures are better able to reflect the specificity of Korean consultative colloquial language.

The introduction of explainable AI (XAI) is also an important achievement of this study. In the medical and mental health sectors, the model “black box” problem has been pointed out as a major barrier to clinical application [30]. Just as Wang et al. secured transparency by applying SHAP to wearable data analysis [31], this study applied SHAP to text data to visualize the basis for the model’s predictions. As a result of the analysis, clinically reasonable keywords such as “I want to die,” “drink,” and “anxiety” showed high importance, indicating that the model learned signs related to actual symptoms rather than data bias (Artifact). Such interpretability serves as an important mechanism for building healthcare professionals’ trust in AI systems [32].

As a result of SHAP-based model interpretation for each category, it was revealed that the core expression that the model focused on when predicting labels consisted of clinically reasonable symptom-related language. In cases classified as depression, expressions indicating despair and sleep problems such as “I want to die” and “I can’t sleep” greatly contributed to model prediction (corresponding to suicidal thoughts and insomnia). In the case of anxiety, expressions related to psychological anxiety and physical tension such as “anxiety” and “heart pounding” acted as important influencers, and in the case of dependence, dependence-related expressions such as “want to quit drinking” and “cravings” were notably used. These expressions correspond to the main symptoms of depression, including suicidal thoughts and sleep disorders; the symptoms of anxiety, including strong physical arousal; and a need for substances, which is a symptom of dependence. In the end, it was found that the handwritten labels in this study were given based on this symptom-related language captured in the consultation conversation rather than a formal diagnostic name, and it was also confirmed that labels assigned at the discretion of experts matched actual language patterns. This supports the clinical validity of this labeling method, and furthermore, it is a result indicating that the characteristics learned by this model are not just frequency-dependent but also linguistic signals based on actual clinical symptoms.

### 4.2. Clinical Implications: Comorbidity Detection

In the clinical practice of mental health, comorbidity is not an exceptional phenomenon, but rather a general rule. A significant number of depression patients worldwide also experience anxiety disorders [33], and the relationship between anxiety and substance use disorders (dependence) has also been fully elucidated. However, there is always a risk that clinicians will overlook potential comorbidities other than a client’s main complaint due to limited hours of care and selective reporting by patients. The multi-label classification model of this study has important significance as an auxiliary tool (decision support system) to bridge such clinical gaps. First, the screening efficiency of dual diagnostics is improved. This model can capture and warn of signs of alcohol dependence and anxiety hidden in the context of a conversation, even if a client mainly complains of depressive symptoms. This contributes to early screening and establishes an integrated treatment plan for patients with depression–addiction comorbidity, which is considered to have a poor treatment outcome. Second, it provides the possibility of objective and constant monitoring. Unlike questionnaires that rely on patient self-reporting, natural language interactive analysis can detect even symptoms that a patient intentionally hides or cannot recognize. For example, when this algorithm is installed in suicide prevention counseling or non-face-to-face mental care platforms, it can be used to classify and prioritize high-risk groups in real time from large-scale counseling data [34].

In addition, the SHAP-based explanatory power shown in this study helps clinicians critically accept AI proposals. If the model classifies a particular patient as part of a “substance use disorder risk group” and presents an utterance that “you must stop drinking” as the basis for this, the counselor can use it as a clue to conduct in-depth interviews. Conversely, it prevents blind AI dependence and promotes human-in-the-loop cooperation because it can reverse-track the cause of its judgment, even if a false positive occurs [35].

In conclusion, this study demonstrated the possibility of a comprehensive screening tool that exceeds the limitations of traditional single disease prediction models by analyzing the interrelated psychopathology of depression, anxiety, and dependence within one integrated framework. Future studies will further improve the accuracy of digital mental health management if they expand to more diverse mental illness categories such as PTSD and schizophrenia and develop into multimodal studies that combine voice and biological signals as well as text.

In summary, four clinical implications follow: (i) intake screening flagging hidden comorbidity that the primary complaint does not surface; (ii) case-conceptualization support SHAP-highlighted utterances act as supervision and training prompts; (iii) CDSS integration embedded in electronic mental health records with dual-level explainability (attention + SHAP) satisfying MFDS 2023 Guidelines on Artificial Intelligence Medical Devices [14]; and (iv) transferability the hierarchical scaffold generalizes to other long-form clinical narratives.

### 4.3. Limitations and Future Work

This study demonstrated the effectiveness of a deep learning model that predicts multiple mental illnesses using counseling dialogue data, but the following limitations exist and need to be supplemented in future studies.

The first is the problem of data imbalance and bias. In all data sets, the number of addiction-positive cases was the lowest at about 26%, so the accuracy of the dependency label (Precision) was measured to be lower than that of other labels. In addition, the model’s ability to detect other behavioral dependencies such as gambling and Internet dependence may be limited because the type of dependence is biased toward alcohol dependence. Future studies need to collect additional cases of various addictions and improve the distribution of data. Technically, data expansion methods using Generative Adversarial Networks (GANs) can be considered as suggested by Shelke and others, but they should be applied carefully to the extent that they do not undermine the natural context of consultative dialogue.

Second, external validity and generalization limitations. This model was trained and verified with only single-domain Korean language counseling data collected by a specific institution. Therefore, when applied to data from other institutions or online communities with different consultation styles or client characteristics, performance degradation may occur. Multi-facility cross-validation is essential in the future to ensure the robustness of the model. In addition, as Cho and others emphasized in a language model review of the medical field, follow-up studies are required to fine-tune the model with new domain data through Transfer Learning and localization and optimization.

Third, a constraint is our single-modality-based approach. Text data conveys the content of the client’s utterance, but does not include nonverbal clues such as voice tone, tremor, speech speed, and facial expression. For example, there is a fundamental limit to fully grasping ironic expressions and subtle changes in emotions such as “I’m really happy to die” through text alone, which can act as an upper limit to model performance (Ceiling). To overcome this, it is necessary to expand to a multimodal deep learning model that combines not only text but also audio signals and image data, such as the Multimodal Hierarchical Attachment (MHA) model proposed by Li et al.

Fourth, it is necessary to deepen interpretability. In this study, the importance of word levels was identified using SHAP, but there is a limit to fully explaining the complex interaction between individual features and time-series contextual effects. For example, it is difficult to interpret the deep reasoning process of the model, whether the word “drink” was simply mentioned or used in the context of dependent needs, with just one value of SHAP. In future studies, attempts to make the model’s decision-making process more three-dimensional and transparent by applying various XAI methods such as time-series SHAP analysis and LIME in a mutually complementary manner are needed.

Finally, there are ethical considerations during actual clinical deployment. AI models have a risk of reproducing biases inherent in learning data, and strict ethical standards are required for personal information protection and microdata handling. This model should be used solely as a decision support system to support expert decision-making, and it should be made clear that the ultimate diagnostic responsibility lies with human experts. In the future, in addition to verifying model performance for real-time streaming data, it will be necessary to upgrade the system, reflecting the latest language trends and clinical situations through continuous online learning.

## 5. Conclusions

Based on actual Korean counseling dialogue data, this study proposed a multilabel classification framework based on hierarchical LSTM and attention, which simultaneously predicts the coexistence of depression, anxiety, and dependence. To overcome the limitation of existing mental health AI studies using binomial classification of single diseases and failing to reflect the complex symptom patterns in actual clinical cases, this study implemented a more comprehensive diagnostic support model through a multi-labeling approach.

The clinical value of the proposed framework lies in its ability to expose hidden comorbidity at intake, to provide token-level rationales that clinicians can audit, and to integrate with electronic mental health records as a transparent decision support tool, in compliance with emerging Korean medical-AI regulation. By coupling hierarchical attention with Deep SHAP and submitting both to independent clinical face-validity review, this work moves AI-assisted mental health screening one step closer to responsible, real-world deployment. If the model proposed by this study is integrated into actual counseling services in the future, it is expected to contribute to the realization of preventive mental health care, which comprehensively manages potential risks that the client is not aware of.

## Figures and Tables

**Table 1:** Data source description.

Item	Description
Source	AI Hub—“Mental Health Counseling Dialogue” dataset (https://aihub.or.kr)
Provider/Governance	National Information Society Agency (NIA), Ministry of Science and ICT, Republic of Korea
Setting	Domestic professional mental health counseling centers (de-identified by data provider)
Collection period	2021–2023
Language	Korean
Modality	Text transcript of client–counselor dyadic dialogue
Sample size	1661 sessions
Average session length	30 min ≈ 5000 Korean characters

**Table 2:** Label and comorbidity distribution across the 1661 counseling sessions.

Configuration	*n*	% of Total
No positive label	242	14.56
Single label—depression only	290	17.46
Single label—anxiety only	297	17.88
Single label—substance use only	268	16.14
Comorbid pair—depression + anxiety	91	4.27
Comorbid pair—depression + substance use	52	3.13
Comorbid pair—anxiety + substance use	36	2.17
Triple comorbidity	27	1.63
Other partial overlaps	378	22.76
Marginal—Depression (any)	484	29.13
Marginal—Anxiety (any)	487	29.31
Marginal—Substance use disorder (any)	448	26.97

**Table 3:** Comparison with prior Korean mental health NLP studies.

Study	Data	Task	Architecture	Explainability	Clinical Val.
Lim & Kwon (2025) [3]	SNS	Single-label	Transformer	-	-
Hsieh et al. (2025) [1]	SNS	Multi-label	LLM	Attention	-
Ji et al. (2022) [23]	Reddit	Pretraining	BERT-domain	-	-
Kerz et al. (2023) [4]	SNS	Single-label	Transformer + XAI	SHAP only	-
This study	Korean counseling	Multi-label comorbidity	HAN-BiLSTM	Attention + DeepSHAP	κ = 0.76

**Table 4:** Performance comparison by model (proposed model HAN-BiLSTM).

Labels	Proposed Model (HAN-BiLSTM) Accuracy	Proposed Model (HAN-BiLSTM) Precision	Proposed Model (HAN-BiLSTM) Recall	Proposed Model (HAN-BiLSTM) F1-Score	Proposed Model (HAN-BiLSTM) AUROC
Depression	0.91	0.88	0.92	0.90	0.95
Anxiety	0.87	0.83	0.87	0.85	0.91
Addiction	0.93	0.72	0.85	0.78	0.87
Micro-Average	0.89	0.85	0.90	0.88	0.91
Macro-Average	0.85	0.81	0.88	0.84	0.91

**Table 5:** Performance comparison by model (criteria model Flat LSTM).

Labels	Baseline Model (Flat LSTM) Accuracy	Baseline Model (Flat LSTM) Precision	Baseline Model (Flat LSTM) Recall	Baseline Model (Flat LSTM) F1-Score	Baseline Model (Flat LSTM) AUC
Depression	0.88	0.85	0.88	0.86	0.92
Anxiety	0.83	0.80	0.82	0.81	0.88
Addiction	0.90	0.65	0.78	0.71	0.80
Micro-Average	0.86	0.81	0.85	0.83	0.80
Macro-Average	0.83	0.77	0.83	0.80	0.87

**Table 6:** Performance comparison by model (reference model KR-BERT).

Labels	Baseline Model (Flat LSTM) Accuracy	Baseline Model (Flat LSTM) Precision	Baseline Model (Flat LSTM) Recall	Baseline Model (Flat LSTM) F1-Score	Baseline Model (Flat LSTM) AUC
Depression	0.90	0.86	0.90	0.88	0.94
Anxiety	0.85	0.78	0.80	0.79	0.86
Addiction	0.91	0.70	0.80	0.75	0.83
Micro-Average	0.88	0.81	0.86	0.83	0.88
Macro-Average	0.85	0.78	0.83	0.8?	0.88

**Table 7:** Confusion matrix by label model component.

	Labels	TP	FP	FN	TN
Proposed Model (HAN-BiLSTM)	Depression	445	61	39	1116
Proposed Model (HAN-BiLSTM)	Anxiety	424	87	63	1087
Proposed Model (HAN-BiLSTM)	Anxiety	381	148	67	1065
Baseline Model (Flat-BiLSTM)	Depression	426	75	58	1102
Baseline Model (Flat-BiLSTM)	Anxiety	399	100	88	1074
Baseline Model (Flat-BiLSTM)	Addiction	349	188	99	1025
Baseline Model (KR-BERT)	Depression	436	71	48	1106
Baseline Model (KR-BERT)	Anxiety	390	110	97	1064
Baseline Model (KR-BERT)	Addiction	358	153	90	1060

**Table 8:** Top SHAP keywords and clinical interpretation (summary).

Label	Top Keywords (Example)	Clinical Interpretation (Summary)
Depression	depressed, struggling, suicide, sad, lonely, tears, exhausted, giving up, lethargic, despair	Depression/lethargy/despair, suicidal ideation and sense of isolation
Anxiety	anxiety, worry, tense, panic, restless, heart pounding, scared, fear, trembling, sensitive	Expressions of worry, tension, panic, and physical arousal (palpitations, tremors)
Addiction	addiction, alcohol, smoking, gambling, gaming, drugs, quit, dependence, treatment, craving	Expressions of loss of control, repetitive behaviors (drinking/gambling/gaming), withdrawal, and craving

**Table 9:** Extended baseline comparison (NEW).

Model	Precision	Recall	F1 (95% CI)	AUROC	*p* vs. Proposed
Flat LSTM	0.77	0.83	0.79 (0.76–0.82)	0.87	<0.001
TextCNN	0.76	0.81	0.78 (0.75–0.81)	0.85	<0.001
KR-BERT	0.78	0.83	0.81 (0.78–0.83)	0.88	<0.001
KoBERT	0.77	0.82	0.79 (0.76–0.82)	0.87	<0.001
KoELECTRA	0.78	0.84	0.81 (0.78–0.83)	0.88	<0.001
KLUE-RoBERTa	0.79	0.85	0.82 (0.79–0.84)	0.89	<0.001
HAN-BiLSTM (ours)	0.81	0.88	0.84 (0.82–0.86)	0.91	—

**Table 10:** Ablation study.

Variant	F1	Δ F1	AUROC
Full HAN-BiLSTM	0.84	—	0.91
(A1)—word-level attention	0.81	−0.028	0.89
(A2)—utterance-level attention	0.78	−0.061	0.86
(A3)—BiLSTM (attention only)	0.80	−0.043	0.87
(A4)—Word2Vec pretraining (random init.)	0.82	−0.022	0.89

## Data Availability

The dataset used in this study is publicly available from the AI Hub “Psychological Counseling Data” dataset (Republic of Korea) at: https://aihub.or.kr/aihubdata/data/view.do?pageIndex=1&currMenu=115&topMenu=100&srchOptnCnd=OPTNCND001&searchKeyword=%EC%8B%AC%EB%A6%AC%EC%83%81%EB%8B%B4&srchDetailCnd=DETAILCND001&srchOrder=ORDER001&srchPagePer=20&aihubDataSe=data&dataSetSn=71806 (accessed on 7 June 2026).
